# Health status assessment of a population of asylum seekers in Northern Italy

**DOI:** 10.1186/s12992-022-00846-0

**Published:** 2022-06-03

**Authors:** Luca Manfredi, Veronica Sciannameo, Cinzia Destefanis, Marta Prisecaru, Giorgia Cossu, Roberto Gnavi, Alessandra Macciotta, Alberto Catalano, Roberto Raffaele Pepe, Carlotta Sacerdote, Fulvio Ricceri

**Affiliations:** 1 Unit of Epidemiology, Regional Health Service ASLTO3, Via Sabaudia 164, 10095 Grugliasco, TO Italy; 2 Department of Biostatistics, Epidemiology and Public Health, Department of Cardiac, Thoracic, Vascular Sciences and Public Health, University of Padova, Padova, Italy; 3 Degree in Strategy and Policy, University of Turin, Turin, Italy; 4 Department of Clinical and Biological Sciences, University of Turin, Turin, Italy; 5 Centre “T. Fenoglio”, Italian Red Cross, Settimo Torinese, TO Italy; 6 Unit of Cancer Epidemiology, Città Della Salute E Della Scienza University-Hospital, Turin, Italy

**Keywords:** Migration, Migrants health, Asylum seekers, Migrants diseases, Migration in Italy

## Abstract

**Background:**

Since 2011 Italy has faced an extraordinary increase in migrants arrivals, mainly from the Mediterranean route, one of the world’s most dangerous journeys. The purpose of the present article is to provide a comprehensive picture of the migrants' health status in the "T. Fenoglio" centre, Settimo Torinese (Turin, Italy).

**Methods:**

A retrospective cross-sectional study was conducted using data collected from June 2016 to May 2018 on adult migrants (over 18 years old) from Africa, Middle East and South East Asia (Bangladesh, Cambodia, India, Nepal). Data was collected through the migrants' medical records. Descriptive statistics were performed on socio-demographic variables. The diagnosed diseases were anonymously registered and classified according to the International Classification of Primary Care (ICPC-2).

Conditional Inference Trees were used to perform a descriptive analysis of the sample and to detect the covariates with the strongest association with the variables Disease on arrival, Disease after arrival, ICPC on arrival and ICPC after arrival.

**Results:**

Analyzed observations were 9 857. 81.8% were men, median age was 23 (Interquartile range: 20.0–27.4). 70.3% of the sample came from Sub-Saharan Africa. 2 365 individuals (24%) arrived at the centre with at least one disease. On arrival, skin (27.71%), respiratory (14.46%), digestive (14.73%) and generic diseases (20.88%) were the most frequent. During the stay respiratory diseases were the most common (25.70%). The highest probability of arriving with a disease occurred in 2018 and during the period September–November 2016, in particular for people from the Horn of Africa. During this period and also in the first half of 2017, skin diseases were the most reported. In seasons with lower prevalence of diseases on arrival the most common disease code was generic for both men and women (usually fever or trauma).

**Conclusions:**

This study provides information on the diverse diseases that affect the asylum seekers population. In our sample, the Horn of Africa was the most troubled area of arrival, with severe conditions frequently reported regarding skin diseases, in particular scabies. 2018 was the most critical year, especially for migrants from the Horn of Africa and Sub-Saharan Africa. During the stay at the camp, the prevalence of respiratory diseases increased. However, skin diseases remained the main issue for people from the Horn of Africa. Overall, the most reported diseases in the sample were dermatological, respiratory, digestive and generic diseases, both on arrival and during the stay.

A better understanding of the health status of asylum seekers is an important factor to determine a more efficient reception and integration process and a better allocation of economic resources in the context of migrants' health care.

**Supplementary Information:**

The online version contains supplementary material available at 10.1186/s12992-022-00846-0.

## Introductions

According to the United Nations High Commissioner for Refugees (UNHCR) (data updated to mid-2020) forcibly displaced people have passed 80 million worldwide, as a result of conflicts, persecutions and other causes. The largest number ever according to available data [1]. Asylum seekers, individuals who are seeking international protection [2], are 16 million over the last decade, two thirds of whom in the last five years [1].

### Migration waves toward Europe

Since the second decade of the century, Europe has faced the greatest migratory challenge since World War II.

A first peak was reached in 2014 with the Ukrainian conflict, while the second in 2015, due to the Syrian war worsening. Moreover, an increasing number of people started to cross the Mediterranean Sea [1].

In the first 10 months of 2020, asylum applications in the EU were 390 000, 33% less compared to the same period of 2019 and significantly lower than the over one million applications registered in 2015 and 2016. According to the European Union Agency for Asylum, the 2020 reduction in applications may be mainly due to Covid-19 pandemic and related measures [3]. The Mediterranean route remains one of the most dangerous, with 1 754 people reported dead or missing in 2020 [4].

The largest group of asylum seekers consists of Syrians in 7 out of 27 EU countries. Three quarters of them are less than 35 years (47% within the range 18–34), 30% are children. Men amount to 61.9% [5].

### The Italian context

The size of the migratory waves, in particular in Italy, has changed over the years.

As a consequence of the Arab Spring (2011), Italy has faced an extraordinary increase in arrivals, mainly from the Mediterranean route (181 000 in 2016). Since 2017 the trend has reversed (119 000), down to 34 154 arrivals in 2020 [6]. Since 2017 the largest number of migrants in Italy came from Nigeria, Guinea, Ivory Coast and Mali. 74% of this population was composed of men. In 2019 the overall number decreased and 21% came from Tunisia (75% men) [6].

The decrease in arrivals may be partly due to several law amendments. Until 2018 the Italian law was based on bilateral agreements with foreign countries, to contain illegal migration. Migrants who entered Italy asking for refugee status were hosted in 19 hubs across the country. In a second stage, they were taken in charge by a second level system (SPRAR: Asylum Seekers and Refugees protection system): applicants for refugee status were given temporary documents, language and professional courses and documentation to work [7]. Over the past few years the Italian legislation has been amended several times [refer to Additional file 1 for details].

### Asylum seekers health profiles and determinants of health

The waves of migration described have posed many challenges to the European health systems. Moreover, there are important differences in the health status among different migrants’ subgroups [8, 9]. In this scenario, more information is needed to better address migrants needs both when they arrive and during their stay in the reception structures.

In addition to the traditional risk factors such as age and gender, the area of origin is likely to affect migrants' health both for endemic situations in the country of origin and for the route to the host country [10–12]. The prevalence of some infectious diseases such as tuberculosis, hepatitis and HIV can reflect the area of origin [10, 11]. Many areas of origin of migrants and refugees have problematic situations regarding low food supply that determines high rates of malnutrition, low vaccine coverage and difficulty in accessing health facilities [11].

On the journey a high percentage of migrants suffer from physical and psychological violence as well as lack of nutrition, medical care and sanitation. They are also exposed to infections and usually have high rates of multimorbidity [11, 13].

The main aim of the present study was to provide a description of the health profile of the asylum seekers hosted in the Piedmont hub, the centre of Settimo Torinese by Turin. Secondly, to analyze factors determining the health status of this population.

## Methods

The present work is a retrospective cross-sectional study on data collected in the period June 1, 2016—May 31, 2018, through medical records of asylum seekers hosted in the "T. Fenoglio" centre, Settimo Torinese (Turin, Italy). The centre has been one of the Italian Red Cross structures involved in the reception plan for asylum seekers as a regional Hub. For unaccompanied minors a different path was determined on arrival.

### Data collection

The centre team was composed of two physicians, a nurse and two psychologists. Weekly visits by specialist physicians were planned. The specialists available for the visits were: cardiologist, dermatologist, gynecologist, infectologist, orthopedic, pneumologist and psychologist. In case of serious health conditions transport to the hospital was provided.

During this time, 15 567 people were admitted to the centre. A medical examination was performed for every subject on arrival. Information about personal data, date of arrival at the centre, date of leaving, health status data, medical history, any diagnoses, treatments and hospitalizations were collected. Moreover, the diseases diagnosed during the first examination or by other exams performed during the stay were anonymously registered and classified according to the International Classification of Primary Care, 2nd edition (ICPC-2) [14]. In case of multimorbidity, up to 3 diseases have been considered.

### Variables of interest

Descriptive statistics were used to present socio-demographic characteristics (Area of origin, Age, Gender, Season) and the distribution of the diseases.

Area of origin was divided into six regions: Sub-Saharan Africa, Northern Africa, Horn of Africa, Central-Southern Africa, Middle East, South East Asia (Bangladesh, Cambodia, India, Nepal) [see Additional file 1 for details]. Age was categorised in classes: 18–24, 25–34, 35 + .

Minors (436, 4.67%) who were present in the centre were only accompanied minors together with the family, as the centre did not host unaccompanied minors. For this reason we excluded them from the analyses as they were not a representative sample of migrant minors. Season groups the time of arrival into seasons: December-February (Winter), March–May (Spring),

June–August (Summer), September–November (Fall), for each available year. Categorical variables were presented as frequencies (n) and percentages (%); continuous ones in terms of medians and interquartile ranges (IQRs).

### Statistical analysis

Conditional Inference Trees were used to perform a descriptive analysis of the sample and to detect the covariates with the strongest association with the outcome variables [see Additional file 1 for algorithm details] [15].

In our study, two of these models were performed for both males and females, in fact the sample has been previously split according to the variable gender, taking into account gender specific diseases. The first outcome variable was Diseases on arrival, a dichotomous variable indicating if the subject has a disease or not. In the second model we used a categorical response variable (ICPC on arrival) having five categories: the four most frequent ICPC-2 classes of diseases (A: generic, D: digestive, R: respiratory, S: skin) and the additional category other, that includes all remaining ICPC-2 codes (in case of multimorbidity the most severe disease was considered). The α level was set to 1%.

The A code states for "General and Unspecified", which includes many diseases from infections like Tuberculosis, Malaria, Measles and Chickenpox to generic and psychological disorders like fever, weakness, body pain, fear of death, fear of various diseases, unspecified trauma, poisoning or adverse effects of medical treatments and others.

### Secondary outcomes and sensitivity analysis

Similar models were performed regarding Diseases after arrival and ICPC-2 after arrival as outcome variables [see Additional file 1].

In addition, Multiple logistic regression models were performed using the same variables to assess trees results [see Additional file 1].

Analyses were conducted using R (V 3.6.2) [16] and SAS (V 9.04).

## Results

### Descriptive statistics

At the time of the analysis many records were found to be incomplete or even missing and the sample was reduced to 9 857 observations. As shown in Table 1, 81.8% of the asylum seekers were men. Median age was 23 years (range: 18–73, IQR: (20.0–27.4)). The most common age group was 18–24 (62.80%).

**Table 1:** Socio-Demographic variables of asylum seekers arrived at the “Fenoglio” centre during the observation period

Variables		Female		Male		Total	
	Categories	n	%	n	%	n	%
Area of origin	Sub-Saharan Africa	1242	72.04%	5687	70.53%	6929	70.31%
	Central- Southern Africa	24	1.39%	39	0.48%	63	0.64%
	Horn of Africa	437	25.35%	1227	15.22%	1664	16.88%
	Middle East	30	1.74%	538	6.67%	568	5.76%
	Northern Africa	60	3.48%	114	1.41%	174	1.77%
	South East Asia	1	0.06%	456	5.66%	457	4.64%
	Total	1794	100%	8063	100%	9855	100%
	missing					2	
Age Group	18–24	1124	62.65%	5066	62.83%	6190	62.80%
	25–34	557	31.05%	2463	30.55%	3020	30.64%
	35 +	113	6.30%	534	6.62%	647	6.56%
	Total	1794	100%	8063	100%	9857	100%
	missing						
Season	June–August 2016	408	22.74%	1368	16.97%	1776	18.02%
	September- November 2016	742	41.36%	3061	37.96%	3803	38.58%
	December 2016- February 2017	146	8.14%	1023	12.69%	1169	11.86%
	March–May 2017	155	8.64%	966	11.98%	1121	11.37%
	June–August 2017	206	11.48%	966	11.98%	1172	11.89%
	September- November 2017	66	3.68%	289	3.58%	355	3.60%
	December 2017- February 2018	57	3.18%	228	2.83%	285	2.89%
	March–May 2018	14	0.78%	153	1.90%	167	1.69%
	Total	1794	100%	8054	100%	9848	100%
	missing					9	

Data collected during the period June 2016-May 2018. Data are presented in terms of frequencies and percentages, as a total and divided by gender. Missing values are indicated, if any. The variable Season refers to the season of arrival. South East Asia: Bangladesh, Cambodia, India, Nepal.

Data was collected since summer 2016. The number of arrivals to the centre was higher in 2016 (max in fall 2016, *n* = 3 803, 38.58%), and gradually decreased during the following years. Length of stay was available for 6 938 observations: median value was 11 days (IQR: 4–28), and 77% of the sample time of stay was less than a month.

Most of the people in the sample came from Africa, in particular from Sub-Saharan Africa (*n* = 6 929, 70.31%). Non-African arrivals were from the Middle East (*n* = 568, 5.76%) and South East Asia (*n* = 457, 4.64%). 2 365 individuals (24%) arrived at the centre with at least one disease, while 1 684 (17.09%) developed at least one during their stay. On arrival, the most frequent diseases were skin (27.71%), respiratory (14.46%), digestive (14.73%) and generic diseases (20.88%) [Table 2], whereas during the stay respiratory diseases considerably increased (25.70%) and a notable decrease occurred for class Skin (12.29%) [Table 3]. Hospitalizations were 232, classified using the ICPC-2 method as well. The most common causes were generic causes (*n* = 35, 15.09%), respiratory diseases (*n* = 28, 12.07%) and pregnancy issues (*n* = 34, 14.66%). Out of the total number of women, pregnancies were 198 on arrival (11.06%). The prevalence of scabies was high for African arrivals: 882 cases detected, of which 39.46% from the Horn of Africa and 57.82% from Sub-Saharan Africa.

**Table 2:** Diseases on arrival, presented in terms of frequencies and percentages (total, by gender and age)

	Female			Male			Total
Classes	18–24	25–34	35 +	18–24	25–34	35 +	
	n (%)	n (%)	n (%)	n (%)	n (%)	n (%)	n (%)
A	56 (20.51)	24 (18.75)	5 (19.23)	248 (22.57)	104 (18.21)	28 (21.54)	465 (20.88)
D	46 (16.85)	11 (8.59)	3 (11.54)	161 (14.65)	95 (16.64)	12 (9.23)	328 (14.73)
R	35 (12.82)	18 (14.06)	4 (15.38)	151 (13.74)	91 (15.94)	23 (17.69)	322 (14.46)
S	70 (25.64)	38 (29.69)	5 (19.23)	329 (29.94)	146 (25.57)	29 (22.31)	617 (27.71)
Other	66 (24.18)	37 (1.56)	9 (34.62)	210 (19.10)	135 (23.64)	38 (29.23)	495 (22.22)

Age groups: 18–24, 25–34, 35 + . The classes refer to the ICPC-2 code

*A* Generic, *D* Digestive, *R* Respiratory, *S* Skin, Other (B: Blood, Blood Forming Organs and Immune Mechanism *F* Eye *H* Hear, *K* Cardiovascular, *L*: Musculoskeletal, *N* Neurological, *P* Psychological. *T* Endocrine/Metabolic and Nutritional, *U* Urological, *W* Pregnancy, Childbearing, Family Planning. X: Female Genital. Y: Male Genital). [see Additional material 1 for details]

**Table 3:** Diseases after arrival, presented in terms of frequencies and percentages (total, by gender and age)

	Female			Male			Total
Classes	18–24	25–34	35 +	18–24	25–34	35 +	
	n (%)	n (%)	n (%)	n (%)	n (%)	n (%)	n (%)
A	66 (16.84)	46 (20.44)	11 (17.46)	246 (23.12)	144 (23.19)	29 (23.19)	513 (21.56)
D	94 (23.98)	45 (20)	6 (9.52)	174 (16.35)	90 (14.49)	17 (11.41)	409 (16.95)
R	67 (17.09)	40 (17.78)	17 (26.98)	289 (27.16)	174 (28.02)	59 (39.60)	587 (25.70)
S	38 (9.69)	21 (9.33)	8 (12.70)	154 (14.47)	77 (12.40)	11 (7.38)	298 (12.29)
Other	127 (32.40)	73 (32.45)	21 (33.34)	201 (18.90)	136 (21.90)	33 (18.42)	558 (23.50)

Age groups: 18–24, 25–34, 35 + . The classes refer to the ICPC-2 code

*A* Generic, *D* Digestive, *R* Respiratory, *S* Skin, Other (B: Blood, Blood Forming Organs and Immune Mechanism. *F* Eye, *H* Hear *K* Cardiovascular *L* Musculoskeletal, *N* Neurological, *P* Psychological, *T* Endocrine/Metabolic and Nutritional, *U* Urological, *W*, Pregnancy, Childbearing, Family Planning, *X* Female Genital, *Y* Male Genital). [see Additional material 1 for details]

### Classification tree models

Classification tree analysis was used to determine the association between the characteristics of the sample (Area of origin, Age, Gender, Season) and two primary outcomes: Diseases on arrival (first tree) and ICPC on arrival (second tree).

#### First tree

**Fig. 1:**
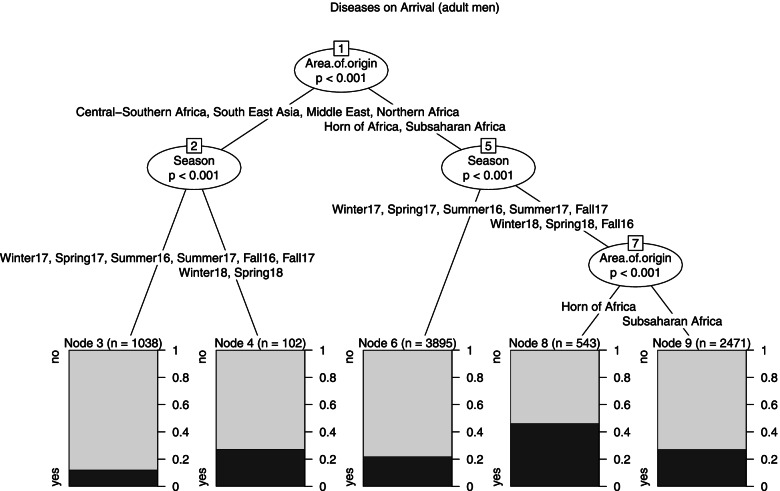
Diseases on arrival, tree model for males

Note: The outcome variable is a binary variable indicating if the subject presented at least one disease on arrival. The predictors are Area of origin (Sub-Saharan Africa, Northern Africa, Horn of Africa, Central-Southern Africa, Middle East, South East Asia (Bangladesh, Cambodia, India, Nepal)), Age (18–24, 25–34, 35 +), Season (December-February (Winter), March–May (Spring), June–August (Summer), September–November (Fall)). The darker side of the leaves represents the probability of arriving with a disease given certain conditions.

Male Model (Fig. 1): The model determined a segmentation into 5 leaves. The strongest associated predictor of Diseases on arrival was Area of origin, with a split between all the areas except the Horn of Africa and Sub-Saharan Africa. Season provided further splits among the subgroups. In the former branch (node 2) the 2018 seasons assessed 27.5% probability of arriving with a disease (node 4, *n* = 102), while the frequency was lower in the previous periods (node 3, *n* = 1 038, 12.2%). In the latter (node 5), a first split was performed between Fall 2016, Winter 2018, Spring 2018 and the other ones (node 6, *n* = 3 895) and that determined a 21.9% probability of arriving with a disease. In node 7 Area of origin determined another split between the Horn of Africa (node 8, *n* = 543) with a 46.2% likelihood of arriving with a disease and Sub-Saharan Africa (node 9, *n* = 2 471) with a likelihood of 27.2%.

**Fig. 2:**
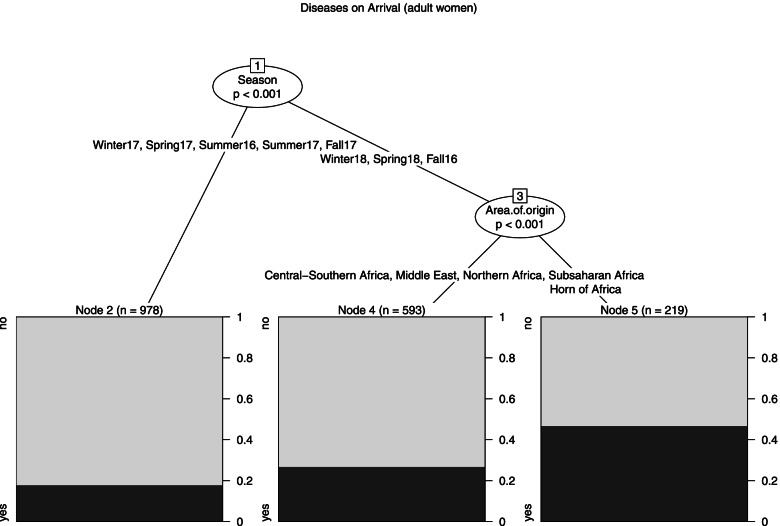
Diseases on arrival, tree model for females

Note: The outcome variable is a binary variable indicating if the subject presented at least one disease on arrival. The predictors are Area of origin (Sub-Saharan Africa, Northern Africa, Horn of Africa, Central-Southern Africa, Middle East, South East Asia (Bangladesh, Cambodia, India, Nepal)), Age (18–24, 25–34, 35 +), Season (December-February (Winter), March–May (Spring), June–August (Summer), September–November (Fall)). The darker side of the leaves represents the probability of arriving with a disease given certain conditions.

Female Model (Fig. 2): The model determined a segmentation into 3 leaves. The strongest predictor of Diseases on arrival was Season: node 2 described the largest subgroup (*n* = 978) of women who arrived in 2017 and Summer 2016. In this case the probability of arriving with a disease was 17.7%. With reference to other seasons (no observations from South East Asia), Area of origin determined a second split between the Horn of Africa (node 5, *n* = 219, probability: 46.6%) and the remaining areas (*n* = 593, probability: 26.6%).

#### Second tree

**Fig. 3:**
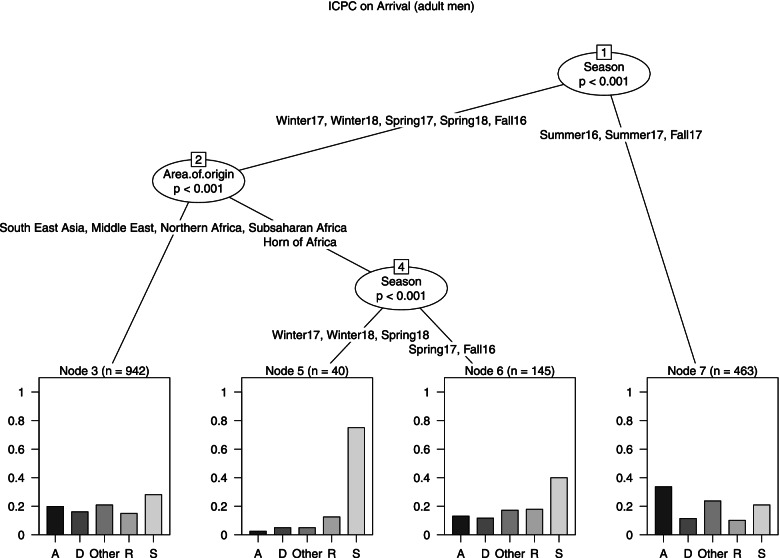
ICPC on arrival, tree model for males who presented a disease on arrival

Note: The outcome variable is a categorical variable having five categories: the four most frequent ICPC-2 classes of diseases (A: generic, D: digestive, R: respiratory, S: skin) and the additional category other, that includes all remaining ICPC-2 codes (in case of multimorbidity the most severe disease was considered). The predictors are Area of origin (Sub-Saharan Africa, Northern Africa, Horn of Africa, Central-Southern Africa, Middle East, South East Asia (Bangladesh, Cambodia, India, Nepal)), Age (18–24, 25–34, 35 +), Season (December-February (Winter), March–May (Spring), June–August (Summer), September–November (Fall)).The darker side of the leaves represents the probability of having a disease of a certain class given certain conditions.

Male Model (Fig. 3): Season resulted to be the strongest predictor of the response variable. The model generated 4 terminal nodes. During Summer 2016 and 2017, Fall 2017 (node 7, *n* = 463) the most frequent diseases were generic ones (33.7%). For the other seasons (no obs. from Central-Southern Africa) a further split was performed by the variable Area of origin: for arrivals from the Horn of Africa, most diseases were included in the category skin (40% in Spring 2017 and Fall 2016, node 6, *n* = 145), particularly in Winters 2017, 2018 and Spring 2018 (node 5, *n* = 40, skin: 75%). Referring to the other areas (node 3, *n* = 942): generic diseases: 19.9%, digestive diseases: 16.1%, other: 20.9%, skin diseases: 28.1%.

**Fig. 4:**
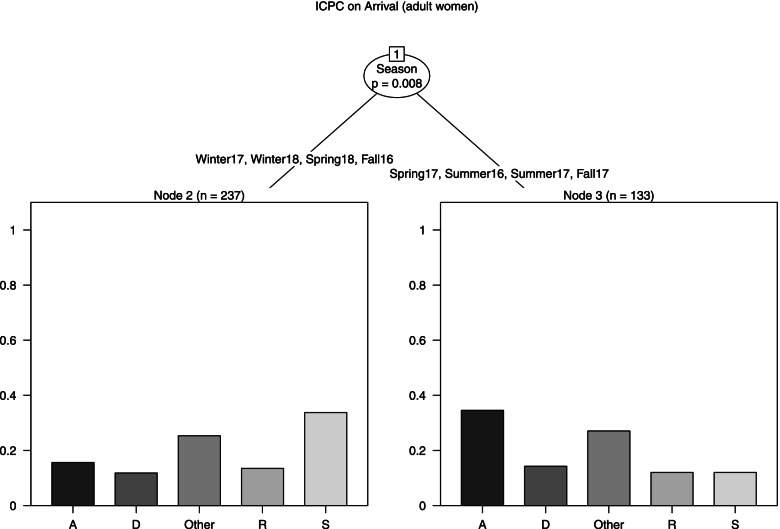
ICPC on arrival, tree model for females who presented a disease on arrival

Note: The outcome variable is a categorical variable having five categories: the four most frequent ICPC-2 classes of diseases (A: generic, D: digestive, R: respiratory, S: skin) and the additional category other, that includes all remaining ICPC-2 codes (in case of multimorbidity the most severe disease was considered). The regressors are Area of origin (Sub-Saharan Africa, Northern Africa, Horn of Africa, Central-Southern Africa, Middle East, South East Asia (Bangladesh, Cambodia, India, Nepal)), Age (18–24, 25–34, 35 +), Season (December-February (Winter), March–May (Spring), June–August (Summer), September–November (Fall)).The darker side of the leaves represents the probability of having a disease of a certain class given certain conditions.

Female Model (Fig. 4): Season was the only predictor of ICPC on arrival. The model performed a single split. For arrivals in Summer 2016 and Spring, Summer and Fall 2017 (node 3, *n* = 133) the most frequent class of diseases was generic (34.6%), followed by other (27.1%). Regarding the other seasons (node 2, *n* = 237), the most frequent classes were skin (33.8%) and other (25.3%). No observations were detected from South East Asia, Middle East and Northern Africa.

## Discussion

The present study describes the health status of asylum seekers hosted in the "T. Fenoglio" centre between June 2016 and May 2018. Areas of arrival and differences in frequency among years reflect the overall history of migration towards Italy.

The asylum seekers were young, mostly men from Sub-Saharan Africa. 24% arrived with at least one disease and the most frequent were dermatological, respiratory, digestive and generic diseases (usually fever, in some cases trauma or not defined conditions) both on arrival and during the stay. Population characteristics and the prevalence of diseases are similar in other studies from countries that received asylum seekers mainly from the “central Mediterranean route” [8, 12, 13, 17–20].

Specifically, we observed that the probability of arriving with a disease was higher in 2018 and Fall 2016, in particular for people from the Horn of Africa. The health conditions worsening in 2018 could be caused by the more and more strict agreements between EU states and Libyan authorities: Italy has been one of the leading countries in assisting the Libyan Coast Guard, in the attempt to limit arrivals. Eventually, Mediterranean departures have decreased since mid-2017, but the mortality rate increased from 1 in 42 in 2017 to 1 in 18 in 2018, according to UNHCR [21]. In the present study the most reported diseases were dermatological, in particular in men arrived from the Horn of Africa, and especially in 2017 and 2018 winters (75%). In seasons with lower prevalence of diseases on arrival the most common code was generic for both men and women.

The asylum seekers from the Horn of Africa have been already recognized as a specific fragile subgroup, especially regarding skin diseases with a high prevalence of scabies [13, 22, 23]. Rate of infection is reported to be even 80% for Eritrean arrivals [24] and 96.2% in a study on orphanages and two refugee camps in Sudan [22]. Moreover, scabies results more prevalent in tropical countries due both to socioeconomic issues (low economic status, domestic crowding) and to the natural developing environment of scabies [25].

Skin diseases in general are caused by distinct factors in different stages of migration. Migrants crossing the Mediterranean Sea by boat are exposed for several days to extreme weather conditions, hygiene and food issues, chemical burns due to the contact with a mix of sea water and petrol. The route from the Horn of Africa is much longer than those from South East Asia and Middle East: according to a survey held in Calais camp, for African refugees the median duration of the journey was 399 days, while it was between 46 and 90 for the other routes [26]. The main difference is determined by the prolonged stay in Libyan hot, airless and overcrowded camps (median time 180 days), where it is virtually impossible to access health care [26–28].

Among the African women hosted at Settimo Torinese Camp, skin diseases were not the major problem, underlining a difference in the distribution of diseases by gender, while classes digestive and other were the most prevalent. In particular, the most common code for class other was female genitals symptoms. Female asylum seekers are often reported as a particularly fragile subpopulation, at high risk of suffering from sexual gender-based violence that cause a higher probability of physical and mental health consequences. This risk is particularly high for routes including Libya. This topic was treated in detail in another study from the same centre [29].

As already observed in different studies that involved mainly arrivals from the Eastern routes toward camps and clinics in Greece (32% Syrians), Brussels (50% from Iraq) and Dresden (classified with ICD-10 code), respiratory diseases are a particular burden for asylum seekers [30–32]. In fact, migrants are vulnerable to antimicrobial resistance, susceptible to influenza for low levels of vaccination, and subject to different pulmonary infections and parasitic diseases such as strongyloidiasis and paragonimiasis. Prevalence in migrants often refers to the endemic situation in countries of origin [33].

A study on migrants hosted in Augusta, Italy (2014) highlighted the heterogeneity of this population by comparing arrivals from the near eastern war zones and Africa. The latter group is composed of young men with higher prevalence of skin diseases (95% of scabies cases out of the total sample). However, respiratory, dermatological, gastrointestinal and trauma conditions had a similar likelihood in both groups [34].

A study on the Castelnuovo di Porto centre (Rome) reported respiratory diseases as the most common, while prevalence of skin diseases was lower. This can be due to the fact that the most represented group came from Pakistan [18].

Finally, for all areas of origin, very low rates in mental illness and psychological distress were observed, despite these being considered a major problem for asylum seekers and migrants in general [10, 33, 35]. Nevertheless, different studies showed a struggle in treating these conditions and an inadequate utilisation of care structures. This is caused by different factors among which many social and cultural barriers [20, 35–38]. In addition, a lower rate in psychological disorders may be due to somatization [20]. Pain general diagnoses (A01 in the ICPC-2 classification) can be brought back to this kind of disorders: in our sample we found 61 cases on arrival (2.88%) and 168 (9.45%) during the stay.

### Strengths and limitations of the study

This study has some strengths: first, it is a unique large collection of health data of asylum seekers once they entered the administrative apparatus for asylum seekers in Italy, one of the countries with the highest rate of immigration. Moreover, the method of classification tree model will help in postulating some reasons for health’ problems, even in the presence of only a little information available.

The study has several limitations: selection bias can be observed because many records were incomplete or missing. Also, the reception path for unaccompanied minors and people victim of violence or abuse was different, and that can partly affect the very low prevalence of mental diseases. The median time of stay was low (10 days) and that can determine an underestimation of diseases after arrival as well as an incorrect estimate of the distribution of different disease types. Moreover, subject data were collected using medical records, so it was impossible to collect information about social determinants of health. In addition, the centre has been closed at the end of 2018, so it was not possible to estimate the impact of COVID-19 pandemic on this population.

## Conclusion

Results on a very large sample of asylum seekers, which contains representatives from 47 countries and all the typical routes towards Italy, were presented. The prevalence and type of diseases observed in the population of asylum seekers of the "T. Fenoglio" centre is similar to other studies which reflect similar areas of origin of the population.

The burden of diseases in the asylum seekers was not a significant threat to public health. In fact, the majority of diseases diagnosed in the population of asylum seekers were mild and not requesting hospitalisation. Anyway, the health surveillance system in asylum seeker centres might necessarily take into account the area of origin, the gender and the season of arrival to organise an adequate and sustainable health care. Finally, a better identification of asylum seekers who need care for mental illness and psychological distress is needed.

The impact of COVID-19 on this population was not assessed, because of the pre-pandemic observation period. An updating of these data with a focus on surveillance activity on COVID-19 and its outcome will be advisable.

## Supplementary Information

**Additional file 1.** Additional information on Italian legislation, tree models algorithm details, Table 2 and Table 3 in details, results of logistic regression analysis to support the main findings.

## Data Availability

For ethical reason, individual data could not be shared. However, aggregated data are available from the corresponding author upon reasonable request.
