# Supplementary material for: Health status assessment of a population of asylum seekers in Northern Italy
Source: Global Health. 2022 Jun 3;18:57. doi: 10.1186/s12992-022-00846-0 (PMC9164173; doi:10.1186/s12992-022-00846-0)
Supplement: Supplementary file 1 — Additional file 1. Additional information on Italian legislation, tree models algorithm details, Table 2 and Table 3 in details, results of logistic regression analysis to support the main findings. [file 12992_2022_846_MOESM1_ESM.docx]

**Supplementary Material**

**1 Introduction**

**Legislation**

**1.1 The European context**

In agreement with the Dublin Regulation if an asylum seeker illegally crosses an EU member state border, the application examination is under responsibility of the first country of entry.[1]

The European Agenda on Migration, adopted in May 2015, provides for the relocation of migrants within the European Union based on a quota system. This system imposes a strict division of migrants into two categories: economic migrants and refugees[1] (people who meet the eligibility criteria under the national legislation[2]).

**1.2 The Italian legislation**

In 2018 the first "Decreto Sicurezza" was issued. It modifies the previous legislation in several ways: the deletion of humanitarian residence permits is determined. In its place the decree introduces a series of special permits. As regards repatriation centres, there is an increase in the maximum time during which foreigners may be detained. The list of offences involving the withdrawal of international protection is being extended and international protection status will be withdrawn if the refugee returns, even temporarily, to his country of origin. The SPRAR (Asylum Seekers and Refugees protection system) has been limited to those who have seen their application for international protection granted, but those who are still applicants, will no longer be able to take part. The latter will then be transferred to other reception centres, where they will wait for decisions on their applications without carrying out particular activities or courses. In 2019 the law was amended with the "Decreto sicurezza bis", which concerns rescue at sea. The law may «restrict or prohibit the entry, transit or stopping of ships in the territorial sea». If a ship breaches the prohibitions imposed, an administrative sanction and the confiscation of the ship, preceded by the immediate seizure, are foreseen. Those caught in the act of resistance or violence against warships must be arrested immediately. In October 2020 the "Decreti sicurezza" were modified by changing the requirements under which international protection will be granted and abolishing the fines against NGOs entering Italian territorial waters after rescuing migrants. The SPRAR system was re-established.[3,4]

**2 Methods**

**2.1 Countries of arrival**

The asylum seekers came from 47 different countries, divided in this article in five macro regions:

Sub-Saharan Africa: Benin, Burkina Faso, Cameroon, Chad, Ivory Coast, Gambia, Ghana, Guinea, Guinea Conakry, Liberia, Mali, Niger, Nigeria, Central African Republic, Senegal, Sierra Leone, Togo.

Horn of Africa: Eritrea, Ethiopia, Somalia, Sudan.

Central-Southern Africa: Burundi, Comoro Islands, Congo, Gabon, Madagascar, Rwanda, South Africa.

Northern Africa: Algeria, Egypt, Libya, Morocco, Mauritania, Tunisia.

Middle East: Afghanistan, Iran, Iraq, Lebanon, Pakistan, Palestine, Syria, Turkey, Yemen.

South East Asia: Bangladesh, Cambodia, India, Nepal.

**2.2 Variables**

Outcome variables: Disease on arrival, Disease after arrival (dichotomous variables indicating if the subject has a disease or not). ICPC on arrival, ICPC after arrival ( categorical response variables having five categories: the four most frequent ICPC-2 classes of diseases (A: generic, D: digestive, R: respiratory, S: skin) and the additional category other, that includes all remaining ICPC-2 codes.[5]

Variables used for the analysis: Area of origin, Age, Season, Gender.

Area of origin was divided into six regions: Sub-Saharan Africa, Northern Africa, Horn of Africa, Central-Southern Africa, Middle East, South East Asia.

Age was categorised in classes: 18-24, 25-34, 35+.

The variable Season groups the time of arrival into seasons: December-February (Winter), March-May (Spring), June-August (Summer), September-November (Fall), for each available year.

**2.3 Trees**

Conditional Inference Trees were used to perform a descriptive analysis of the sample and to detect the covariates with the strongest association with the outcome variables. This class of non-parametric trees, unlike most recursive partitioning algorithms, takes into account the distributional properties of the measures. In fact, the simple recursive partitioning algorithms may suffer overfitting and selection bias of the covariates, when those allow for many splits to choose from. Moreover, multiple test procedures are applied to determine whether no significant association between any of the covariates and the response can be stated and the recursion needs to stop.

Given a sample of n observations L_n_, a vector of weights w = (w_1_,...,w_n_) is associated to the observations, so that every node is represented by a vector of weights | w_i_ = 1, i = 1, ..., n if the corresponding observation belongs to the node, w_i_ = 0 otherwise.

For each node w, the global null hypothesis H_0_ is built in terms of m partial hypotheses H_0_^j^,j=1,...,m: D(Y|X_j_)=D(Y) => H_0_ = ∩^m^_j=1_H_0_^j^. If the global H_0_ is rejected associations are measured by statistics that measure deviation from H_0_^j^.

Algorithm steps:

1. Given the vector of weights w, the algorithm tests H_0_, the global null hypothesis of independence between Y and any of the m covariates. If the hypothesis is rejected, X_j_ is selected so that it has the strongest association with Y.
2. A subset A ⊂ χ _j_ is chosen ( where χ _j_ is the space of the covariate X_j_) | χ _j_ is split into A and χ _j_/A.
3. w_R_ and w_L_ determine the subgroups with
   w_Li_ = w_i_I(X_ji_ ∈ A), w_Ri_ = w_i_I(X_ji_ ∈ χ _j_ / A), i = 1,...,n.
4. Steps 1. and 2. are recursively repeated, using the new weights w_R,_ w_L_

The algorithm stops when the global H_0_ of independence between Y and any of the covariates cannot be rejected for a certain level α.

Moreover, Bonferroni correction for adjusted p-values was used to reduce the probability of type I error in testing the multiple partial null hypotheses that define H_0_. The previous steps lead to a partition of χ such that each part B_i_ is associated with a vector of weights w. Compared to the more common recursive partitioning algorithms, the variable and cutpoints choices are made in two different steps and this allows to avoid the tendency to select variables with many possible splits.[6]

**3 Results**

**3.1 Trees for Diseases after arrival**

**3.1.1 Third Tree**

**Male Model** (Figure 5): The model determined a segmentation into 4 leaves. The strongest associated predictor was Season, with a split between 2017, Spring 2018 (node 2) and the remaining quarters. For the last subgroup (node 7, n=4 656) the likelihood of developing a disease during the stay was very low (5.7%). Season provided further splits in the first subgroup: Winter and Spring 2017 (node 3), and Summer and Fall 2017, Spring 2018 (node 6, n=1 406) with a 22.8% probability of developing a disease during the stay. The former subgroup has been split again according to the variable Area of origin, determining two terminal nodes: the sample from Central-Southern Africa, Horn of Africa and Northern Africa had a probability of 17.8% of developing a disease (node 4, n=208), whereas for arrivals from South East Asia, Middle East and Sub-Saharan Africa the prevalence was greater (node 5, n=1 781, 36.4%).

**[Figure 5.**  Diseases after arrival, tree model for males.

Note: The outcome variable is a binary variable indicating if the subject developed at least one disease after arrival. The predictors are Area of origin (Sub-Saharan Africa, Northern Africa, Horn of Africa, Central-Southern Africa, Middle East, South East Asia (Bangladesh, Cambodia, India, Nepal)), Age (18-24, 25-34, 35+), Season (December-February (Winter), March-May (Spring), June-August (Summer), September-November (Fall)). The darker side of the leaves represents the probability of developing a disease given certain conditions.]

**Female Model** (Figure 6): The splitting procedure resulted in 5 leaves and Season as the strongest predictor. The lowest probability (5.1%) is observed for the Summer 2016 subgroup (node 6, n=408). In the remaining 2016 quarter the likelihood for women of age 35 and more was 36.1% (node 9, n=36) while for the other age groups node 8 determined a probability equal to 11.9% (node 8, n=706). In 2017 and 2018, a 50.9% likelihood was found for the Sub-Saharan area (node 4, n=491). 30.1% for the other areas (node 3, n=153).

**[Figure 6.** Diseases after arrival, tree model for females.

Note: The outcome variable is a binary variable indicating if the subject developed at least one disease after arrival. The predictors are Area of origin (Sub-Saharan Africa, Northern Africa, Horn of Africa, Central-Southern Africa, Middle East, South East Asia (Bangladesh, Cambodia, India, Nepal)), Age (18-24, 25-34, 35+), Season (December-February (Winter), March-May (Spring), June-August (Summer), September-November (Fall)). The darker side of the leaves represents the probability of developing a disease given certain conditions.]

**3.1.2 Fourth Tree**

**Male Model** (Figure 7): The algorithm generated a tree with 4 terminal nodes. The strongest predictor was Area of origin. The Horn of Africa has been split from the other areas, and this subgroup (node 7, n=106) determined a 32.1% probability of developing a Skin disease, while probabilities for other classes were lower. From other areas, the subgroup that arrived during Summer 2016, Spring, Summer and Fall 2017, Winter 2018 (node 6, n=639) had Generic as the most frequent class (28.2%). For other quarters, a further split was performed according to the variable Area of origin: for arrivals from the Middle East (node 5, n=65) over half post-arrival diseases were of type Respiratory (58.5%). For the remaining areas (node 4, n=432) the most frequent class was Respiratory as well (32.2%), with all other classes around 17%.

**[Figure 7.**  ICPC after arrival, tree model for males who developed a disease during the stay.

Note: The outcome variable is a categorical variable having five categories: the four most frequent ICPC-2 classes of diseases (A: Generic, D: Digestive, R: Respiratory, S: Skin) and the additional category other, that includes all remaining ICPC-2 codes (in case of multimorbidity the most severe disease was considered). The predictors are Area of origin (Sub-Saharan Africa, Northern Africa, Horn of Africa, Central-Southern Africa, Middle East, South East Asia (Bangladesh, Cambodia, India, Nepal)), Age (18-24, 25-34, 35+), Season (December-February (Winter), March-May (Spring), June-August (Summer), September-November (Fall)). The darker side of the leaves represents the probability of having a disease of a certain class given certain conditions.]

**Female Model** (Figure 8): In the last model, the only associated variable (Season) carried out a single split generating two terminal nodes. For Summer 2016, Fall 2017, Spring 2018 (node 3, n=54) the most frequent class was Digestive (40.7%), followed by class other (24.1%), Generic (18.5%), Respiratory (11.1%) and Skin (5.6%). In node 2 (n=355) class other had the highest probability (30.1%) (Respiratory: 23.1%, Generic: 17.7%, Digestive: 16.6%, Skin: 12.4%).

**[Figure 8.**  ICPC after arrival, tree model for females who developed a disease during the stay.

Note: The outcome variable is a categorical variable having five categories: the four most frequent ICPC-2 classes of diseases (A: Generic, D: Digestive, R: Respiratory, S: Skin) and the additional category other, that includes all remaining ICPC-2 codes (in case of multimorbidity the most severe disease was considered). The predictors are Area of origin (Sub-Saharan Africa, Northern Africa, Horn of Africa, Central-Southern Africa, Middle East, South East Asia (Bangladesh, Cambodia, India, Nepal)), Age (18-24, 25-34, 35+), Season (December-February (Winter), March-May (Spring), June-August (Summer), September-November (Fall)). The darker side of the leaves represents the probability of having a disease of a certain class given certain conditions.]

**3.2 Logistic regressions**

Multivariate logistic regression models were performed to assess association between different factors and the binary outcome variables Diseases on arrival and Diseases after arrival. These models were built in order to compare the tree algorithms with a more commonly used technique. The estimates shown in table 4 and table 5 are consistent with the trees results.

**4 References**

1. Migration and Home affairs. European Commission. Available at: <https://ec.europa.eu/home-affairs/what-we-do/policies/asylum/examination-of-applicants_en>
2. UN High Commissioner for Refugees (UNHCR), UNHCR Master Glossary of Terms, June 2006, Rev.1
3. Le leggi italiane sull’asilo. UNHCR Italia. Available at: <https://www.unhcr.org/it/cosa-facciamo/protezione/diritto-asilo/> italia/legislazione/
4. Vita. Superati i decreti Salvini, il nuovo testo su sicurezza e immigrazione. Available at:[http://www.vita.it/it/article/2020/10/06/superati-i-decreti-salvini-il\](http://www.vita.it/it/article/2020/10/06/superati-i-decreti-salvini-il%5C) -nuovo-testo-su-sicurezza-e-%20immigrazione/156889/
5. International Classification of Primary Care, 2nd edition (ICPC-2). WHO. Available at: <https://www.who.int/standards/classifications/other-classifications/>international-classification-of-primary-care
6. Hothorn T., Hornik K., Zeileis A. (2006). Unbiased Recursive Partitioning: A Conditional Inference Framework. Available at: <https://www.zeileis.org/papers/Hothorn+Hornik+Zeileis-2006.pdf>

**Table2.** Diseases on arrival, presented in terms of frequencies and percentages (total, by gender and age).

|  | Female |  |  | Male |  |  | Total |
| --- | --- | --- | --- | --- | --- | --- | --- |
| Classes | 18-24 | 25-34 | 35+ | 18-24 | 25-34 | 35+ |  |
|  | n  (%) | n  (%) | n  (%) | n  (%) | n  (%) | n  (%) | n  (%) |
| A | 56  (20.51) | 24  (18.75) | 5  (19.23) | 248 (22.57) | 104 (18.21) | 28  (21.54) | 465 (20.88) |
| B | 5  (1.83) | 1  (0.78) | . | 7  (0.64) | 7  (1.23) | . | 20  (0.90) |
| D | 46  (16.85) | 11 (8.59) | 3  (11.54) | 161 (14.65) | 95  (16.64) | 12  (9.23) | 328 (14.73) |
| F | 4  (1.47) | 1  (0.78) | 1  (3.85) | 24  (2.18) | 11  (1.93) | 2  (1.54) | 43  (1.93) |
| H | . | . | . | 13  (1.18) | 4  (0.70) | 1  (0.77) | 18  (0.81) |
| K | 2  (0.73) | 5  (3.91) | . | 15  (1.36) | 14 (2.45) | 7  (5.38) | 43  (1.93) |
| L | 6  (2.20) | 18  (14.06) | 4  (15.38) | 81  (7.37) | 55  (9.63) | 17  (13.08) | 181  (8.813) |
| N | 12  (4.40) | 3  (2.34) | 2  (7.69) | 29  (2.64) | 12  (2.10) | 5  (3.85) | 63  (2.83) |
| P | 4  (1.47) | 1  (0.78) | . | 7  (0.64) | 11  (1.93) | 1  (0.77) | 24  (1.08) |
| R | 35  (12.82) | 18  (14.06) | 4  (15.38) | 151 (13.74) | 91  (15.94) | 23  (17.69) | 322 (14.46) |
| S | 70  (25.64) | 38  (29.69) | 5  (19.23) | 329 (29.94) | 146 (25.57) | 29  (22.31) | 617 (27.71) |
| T | . | 1  (0.78) | 2  (7.69) | 1  (0.09) | 3  (0.53) | 3  (2.31) | 10  (0.45) |
| U | 2  (0.73) | 1  (0.78) | . | 9  (0.82) | 9  (1.58) | . | 21  (0.94) |
| W | 2  (0.73) | . | . | . | . | . | 2  (0.09) |
| X | 29  (10.62) | 6  (4.69) | . | . | . | . | 35  (1.57) |
| Y | . | . | . | 24  (2.18) | 9  (1.58) | 2  (1.54) | 35  (1.57) |

Note: Age groups: 18-24, 25-34, 35+. The classes refer to the ICPC-2 code. A: Generic. B: Blood, Blood Forming Organs and Immune Mechanism. D: Digestive. F: Eye. H: Hear. K: Cardiovascular. L: Musculoskeletal. N: Neurological. P: Psychological. R: Respiratory. S: Skin. T: Endocrine/Metabolic and Nutritional. U: Urological. W: Pregnancy, Childbearing, Family Planning. X: Female Genital. Y: Male Genital.

**Table 3.** Diseases after arrival, presented in terms of frequencies and percentages (total, by gender and age).

|  | Female |  |  | Male |  |  | Total |
| --- | --- | --- | --- | --- | --- | --- | --- |
| Classes | 18-24 | 25-34 | 35+ | 18-24 | 25-34 | 35+ |  |
|  | n  (%) | n  (%) | n  (%) | n  (%) | n  (%) | n  (%) | n  (%) |
| A | 66  (16.84) | 46  (20.44) | 11  (17.46) | 246 (23.12) | 144 (23.19) | 29  (23.19) | 513  (21.56) |
| B | . | . | . | 1  (0.09) | 1  (0.16) | 1  (0.67) | 2  (0.12) |
| D | 94  (23.98) | 45  (20) | 6  (9.52) | 174 (16.35) | 90  (14.49) | 17  (11.41) | 409 (16.95) |
| F | 5  (1.28) | 4  (1.78) | 3  (4.76) | 18  (1.69) | 18  (2.90) | 5  (3.36) | 48  (2.11) |
| H | 6  (1.53) | 2  (0.89) | 1  (1.59) | 9  (0.85) | 9  (1.45) | . | 27  (1.07) |
| K | 1  (0.26) | 2  (0.89) | 2  (3.17) | 6  (0.56) | 8  (1.29) | . | 19  (0.76) |
| L | 8  (2.04) | 13  (5.78) | 6  (9.52) | 60  (5.64) | 45  (7.25) | 9  (6.04) | 132  (5.61) |
| N | 27  (6.89) | 17  (7.56) | 4  (6.35) | 66  (6.20) | 32  (5.15) | 10  (6.71) | 146  (6.21) |
| P | 7  (1.79) | 3  (1.33) | 2  (3.17) | 12  (1.13) | 5  (0.81) | 2  (1.34) | 29  (1.23) |
| R | 67  (17.09) | 40  (17.78) | 17  (26.98) | 289 (27.16) | 174 (28.02) | 59  (39.60) | 587  (25.70) |
| S | 38  (9.69) | 21  (9.33) | 8  (12.70) | 154 (14.47) | 77  (12.40) | 11  (7.38) | 298  (12.29) |
| T | 4  (1.02) | . | 2  (3.17) | 1  (0.89) | 1  (0.16) | 1  (0.67) | 8  (0.36) |
| U | 12  (3.06) | 5  (2.22) | . | 18  (1.69) | 13  (2.09) | 3  (2.01) | 48  (2.03) |
| W | 11  (2.81) | 5  (2.22) | . | . | . | . | 16  (0.64) |
| X | 46  (11.73) | 22  (9.78) | 1  (1.59) | . | . | . | 69  (2.74) |
| Y | . | . | . | 10  (0.94) | 4  (0.64) | 2  (1.34) | 14  (0.64) |

Note: Age groups: 18-24, 25-34, 35+. The classes refer to the ICPC-2 code. A: Generic. B: Blood, Blood Forming Organs and Immune Mechanism. D: Digestive. F: Eye. H: Hear. K: Cardiovascular. L: Musculoskeletal. N: Neurological. P: Psychological. R: Respiratory. S: Skin. T: Endocrine/Metabolic and Nutritional. U: Urological. W: Pregnancy, Childbearing, Family Planning. X: Female Genital. Y: Male Genital.

**Table 4.** Logistic regression analysis on the full sample. The outcome variable is Diseases on arrival.

| Variable |  | OR estimate | Lower 95% CL | Upper 95% CL |
| --- | --- | --- | --- | --- |
|  | Categories |  |  |  |
| Age (ref:18-24) | 25-34 | 1.082 | 0.976 | 1.200 |
|  | 35+ | 1.106 | 0.906 | 1.349 |
|  |  |  |  |  |
| Sex (ref:Male) | Female | 0.910 | 0.805 | 1.029 |
|  |  |  |  |  |
| Season (ref:Q3/16) | Fall 2016 | 1.447 | 1.265 | 1.654 |
|  | Winter 2017 | 0.847 | 0.698 | 1.028 |
|  | Spring 2017 | 0.966 | 0.802 | 1.164 |
|  | Summer 2017 | 0.858 | 0.713 | 1.032 |
|  | Fall 2017 | 0.993 | 0.739 | 1.335 |
|  | Winter 20178 | 2.001 | 1.510 | 2.650 |
|  | Spring 2018 | 2.074 | 1.486 | 2.893 |
|  |  |  |  |  |
| Area of origin (ref:  Sub-Saharan Africa) | Central-Southern Africa | 0.415 | 0.188 | 0.916 |
|  | Horn of Africa | 1.486 | 1.317 | 1.676 |
|  | Northern Africa | 0.493 | 0.312 | 0.779 |
|  | Middle East | 0.512 | 0.396 | 0.662 |
|  | South East Asia | 0.482 | 0.333 | 0.587 |

Note: The variable Season refers to the season of arrival. December-February (Winter), March-May (Spring), June-August (Summer), September-November (Fall). South East Asia: Bangladesh, Cambodia, India, Nepal.

**Table 5.** Logistic regression analysis on the full sample. The outcome variable is Diseases after arrival.

| Variable |  | OR estimate | Lower 95% CL | Upper 95% CL |
| --- | --- | --- | --- | --- |
|  | Categories |  |  |  |
| Age (ref:18-24) | 25-34 | 1.297 | 1.144 | 1.469 |
|  | 35+ | 1.485 | 1.185 | 1.859 |
|  |  |  |  |  |
| Sex (ref:Male) | Female | 2.233 | 1.937 | 2.575 |
|  |  |  |  |  |
| Season (ref:Q3/16) | Fall 2016 | 1.567 | 1.215 | 2.021 |
|  | Winter 2017 | 11.077 | 8.549 | 14.352 |
|  | Spring 2017 | 13.851 | 10.713 | 17.907 |
|  | Summer 2017 | 7.701 | 5.943 | 9.979 |
|  | Fall 2017 | 9.766 | 7.053 | 13.515 |
|  | Winter 2018 | 3.711 | 2.468 | 5.580 |
|  | Spring 2018 | 7.268 | 4.744 | 11.135 |
|  |  |  |  |  |
| Area of origin  (ref:  Sub-Saharan Africa) | Central-Southern Africa | 0.369 | 0.167 | 0.816 |
|  | Horn of Africa | 0.602 | 0.501 | 0.725 |
|  | Northern Africa | 0.400 | 0.260 | 0.615 |
|  | Middle East | 0.685 | 0.537 | 0.615 |
|  | South East Asia | 0.678 | 0.499 | 0.921 |

Note: The variable Season refers to the season of arrival. December-February (Winter), March-May (Spring), June-August (Summer), September-November (Fall). South East Asia: Bangladesh, Cambodia, India, Nepal.
